# Characterization of Cyanophages in Lake Erie: Interaction Mechanisms and Structural Damage of Toxic Cyanobacteria

**DOI:** 10.3390/toxins11080444

**Published:** 2019-07-26

**Authors:** Xuewen Jiang, Chanhee Ha, Seungjun Lee, Jinha Kwon, Hanna Cho, Tyler Gorham, Jiyoung Lee

**Affiliations:** 1 Department of Food Science and Technology, The Ohio State University, Columbus, OH 43210, USA; 2 Department of Mechanical and Aerospace Engineering, The Ohio State University, Columbus, OH 43210, USA; 3 College of Public Health, Division of Environmental Health Sciences, The Ohio State University, Columbus, OH 43210, USA

**Keywords:** *Podoviridae*, atomic force microscopy, mechanical stiffness, *Microcystis*, harmful algal bloom

## Abstract

Cyanophages are abundant in aquatic environments and play a critical role in bloom dynamics, including regulation of cyanobacteria growth and photosynthesis. In this study, cyanophages from western Lake Erie water samples were screened for lytic activity against the host cell (*Microcystis aeruginosa*), which also originated from Lake Erie, and identified with real-time sequencing (Nanopore sequencing). *M. aeruginosa* was mixed with the cyanophages and their dynamic interactions were examined over two weeks using atomic force microscopy (AFM) as well as transmission electron microscopy (TEM), qPCR, phycocyanin and chlorophyll-a production, and optical absorbance measurements. The TEM images revealed a short-tailed virus (*Podoviridae*) in 300 nm size with unique capsid, knob-like proteins. The *psbA* gene and one knob-like protein gene, *gp58*, were identified by PCR. The AFM showed a reduction of mechanical stiffness in the host cell membranes over time after infection, before structural damage became visible. Significant inhibition of the host growth and photosynthesis was observed from the measurements of phycocyanin and chlorophyll-a concentrations. The results provide an insight into cyanobacteria–cyanophage interactions in bloom dynamics and a potential application of cyanophages for bloom control in specific situations.

## 1. Introduction

Cyanobacterial blooms in freshwater have been a growing concern not only in the United States but also globally, with increasing frequency, duration, and intensity [1]. They pose a great threat for environmental and public health because of toxic compounds released from the blooms, and cause significant economic loss for those bloom-affected areas [2,3,4,5]. These toxins, such as microcystins, saxitoxins, nodularins and cylindrospermopsin, are widely distributed across the world and are difficult to remove [3]. Previous studies focused on controlling blooms in many ways, including applying various chemicals [6,7] and controlling eutrophication of waterbodies [8,9]. However, more specific and targeted approach is needed for controlling toxic blooms without adding more chemicals.

Cyanophages are viruses that infect cyanobacteria as their host. Similar to other bacteriophages, cyanophages can alter the metabolism and the replication of their hosts, then further influencing the structure of the cyanobacterial community [10,11,12]. The succession of toxic *Microcystis aeruginosa*, one of the most commonly found toxic cyanobacteria in freshwater, is affected by the abundance of its cyanophages, indicating that cyanophages may play a critical role in bloom formation dynamics [13,14,15]. Therefore, cyanophages were considered as a potential biological control of cyanobacterial blooms [16,17]. Bacteriophages are promising for controlling bacterial infections, with great advantages of its host specificity. Instead of using chemicals, such as antibiotics, bacteriophages would minimize the side effect of disturbing other natural microbiota [18,19].

While most freshwater cyanophages are known as tailed *Myoviridae* (with a long contractile tail), *Podoviridae* (short non-contractile tail), *Siphoviridae* (long non-contractile tail), and tail-less phages have been also reported [20,21,22,23,24,25]. Current PCR-based diagnoses of cyanophages are mostly targeting structural genes of *Myoviridae*, such as capsid protein gene (*g20*) and tail sheath protein (*g91*) [15,26,27,28,29]. The presence and abundance of other cyanophage families in environments are still highly underestimated [29]. In addition, active gene exchanges between cyanophages and their hosts make it more difficult to find a quantitative target.

At a morphological level, the development of microscopy with high-resolution, three-dimensional imaging, such as atomic force microscopy (AFM), enables a more accurate description of host–phage interactions. Previous studies described the application of AFM as a versatile tool to explore phage infections from a morphological to a molecular scale [30,31]. To achieve high sensitivity on soft cyanobacteria samples, AFM’s tapping mode was performed. For this, an AFM probe, consisting of a micro-cantilever with a nanometer scale tip, was driven to oscillate at/or near its resonance frequency and gently tap the sample surface that was characterized. Then the amplitude and phase of oscillations were changed by the tip–sample interactions, which were measured by a laser detection system. The recorded change in amplitude provides the morphology of the sample, while the change of phase reveals the compositional variations. It has recently been applied for observing viruses, including cyanophages [31,32].

The main objectives of this study were to: (1) characterize lytic cyanophages isolated from Lake Erie, both morphologically and genetically; and (2) examine the host (*M. aeruginosa*) and cyanophage interactions using multiple tools, including signature gene screening, photopigment measurements, transmission electron microscopy (TEM), and AFM. Thus, we aimed to gain insights into potential use of cyanophages for controlling toxic cyanobacteria proliferation under applicable situations.

## 2. Results and Discussion

### 2.1. Screening of Lytic Cyanophages

Water samples from western Lake Erie were collected from May to August 2015 and were screened for cyanophages. Among the samples, the cyanophage with the highest lytic activities against *M. aeruginosa* was selected. It was named as Ma-LEP, which indicates its host strain (*Microcystis aeruginosa,* Ma), origin (Lake Erie, LE) and taxonomic family (*Podoviridae*, see below for more details). The host, *Microcystis aeruginosa,* was also isolated from Lake Erie and confirmed with PCR by targeting PC-IGS and toxin-producing genes (*mcyA*, and *mcyE*), and identified with real-time sequencing technique (MinION, Oxford Nanopore Technologies) (Figure S1).

### 2.2. The Effects of Ma-LEP Infection on Microcystis aeruginosa

**Figure 1:**
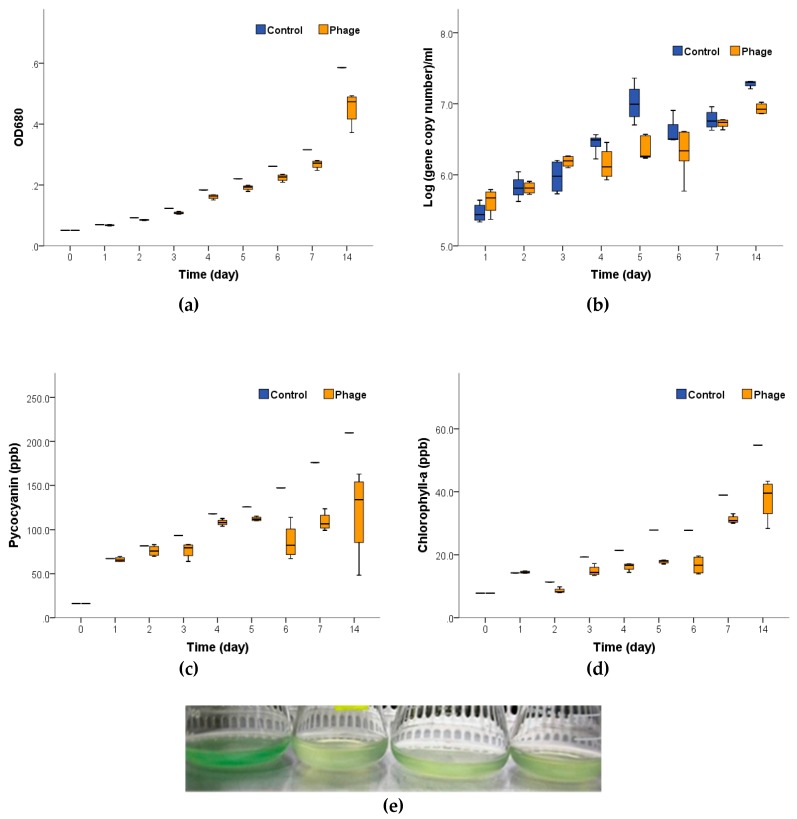
The dynamic changes of *M. aeruginosa* infected by cyanophage Ma-LEP in two weeks. (**a**) optical density (OD) at 680 nm; (**b**) the concentration of *M. aeruginosa* (*mcyE* gene); (**c**) the concentration of phycocyanin; (**d**) the concentration of chlorophyll-a.; (**e**) control (*M. aeruginosa* only, left) and infected by cyanophage Ma-LEP (right three).

To examine effects of lytic cyanophage Ma-LEP on *M. aeruginosa* (host), dynamic change of host population was measured with multiple parameters; OD (680 nm), two important photosynthetic pigments (phycocyanin and chlorophyll-a) and targeted gene of *M. aeruginosa* (*mcyE*). Figure 1 shows the dynamic changes of these parameters over time after phage infections. Table 1 summarizes the growth of pigment production rate (slope) and the correlation coefficient (*R*^2^) of each parameter when fitting the raw data of Figure 1 in linear models (for *mcyE* gene, data from day 0 to day 5 had a strong fit of the linear regression).

Ma-LEP impaired the growth and photosynthesis of *M. aeruginosa* significantly when compared with the control group (*M. aeruginosa* host inoculated with autoclaved cyanophage), indicating that Ma-LEP can slow down or reduce bloom intensity. The phage-infected *M. aeruginosa* showed a significant decrease (*p* < 0.01) in their growth rates (OD at 680 nm, Figure 1a). The qPCR results (Figure 1b) showed the *mcyE* gene concentration (microcystin-producing *M. aeruginosa* abundance) was reduced at days 4, 5, and 14 (*p* < 0.05). Noteworthy, the extracellular DNA from lysed cells was also contributing to the total DNA copy counts; therefore, using qPCR might have overestimated the host count, especially in the cyanophage group. Meanwhile, the production of the photosynthetic pigments, phycocyanin, and chlorophyll-a, was significantly reduced (*p* < 0.01) after Ma-LEP infection, possibly due to the cell lysis or suppression of related genes by cyanophages (Figure 1c,d). For more accurate measurements of *M. aerguginosa*’s photosynthetic activities, amount of carbon fixed, and oxygen production per unit time should be measured.

In addition, Ma-LEP also showed lysogenic activity during continuous culturing under lab conditions when appropriate dose of UV light was applied. It was found that 23.58 mJ/cm^2^ of UV intensity was sufficient for the cyanophage to activate cell lysis (Figure S2; Table S1).

Since the toxin concentrations were the sum of both free (released from dead cells) and particulate (from intact cells) toxins within the confined flasks, no statistical significance was observed in microcystin concentrations between the two groups (phage treated vs. control) during the one-week period (Figure S3).

**Figure 2:**
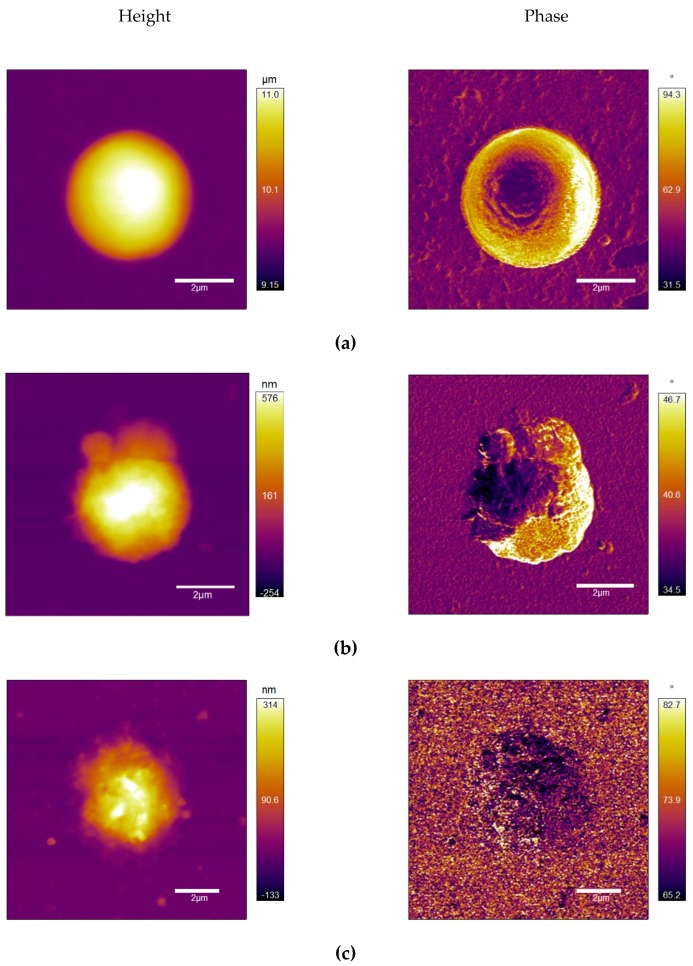
Structural changes of *M. aeruginosa* caused by cyanophage Ma-LEP infection. (**a**) Control group; (**b**,**c**) cyanophage Ma-LEP infected group. Left and right columns show the morphological change and the stiffness damage of the host cells, respectively, as the infection progressed.

AFM is an emerging technique in biological studies, including cells, viruses, and biological molecules (DNA, protein, etc.) [1,2,3,4,5]. In this study, AFM images provided an in-depth view of the physical changes of infected host. To better visualize the host–phage interaction over time, an air tapping mode of AFM was used. It applied a fine tip to the object surface and monitored the frequency change of the tip when it interacted with different shapes, material, etc. The height images (the left column in Figure 2 provides morphological information of the cyanobacterial hosts) and the phase images (the right column in Figure 2) show the changes in stiffness of the targeted objects (*M. aeruginosa*). The changes from both images revealed the structural damages of *M. aeruginosa* cells after the cyanophage Ma-LEP infection. Initially, the *M. aeruginosa* was observed in a clear circular shape about 2 μm tall in contrast to the background (mica, in this case), indicating the intactness of host cells (Figure 2a). Following cyanophage infection, cells became irregular and started to shrink and break down, while the height was reduced to 0.8 and 0.4 μm (Figure 2b,c, only one representative cell was shown in Figure 2) and, finally resulted in the rupture of the entire cell. Actually, the tapping mode can achieve resolution down to several nanometers without damaging the samples (either during sample preparation or imaging steps), allowing repeated observations and flexible applications [6]. Therefore, AFM air tapping modes can be used repeatedly to further observe viral topology (e.g., the arrangement of the “knob-like protein” on Ma-LEP capsids). AFM also enables observation of biological specimens in fluid, which can maintain the bioactivity of samples and allows more vivid visualization [1]. This method allows capturing the cellular changes of hosts at lytic cycle *in vivo*, which may provide more information on host–phage interactions and mechanisms of interests in future studies.

The interpretation of phase images requires a detailed understanding about the cantilever dynamics depending on the AFM tip–sample interactions. When the AFM tip hovers over a sample instead of indenting it due to relatively high attractive forces (so-called attraction-dominant regime), the phase becomes larger than 90°. In the phase map of Figure 2a, the perimeter of the cyanobacteria showing the bright color over 90° indicates that the tip experiences a strong attractive force. We conjecture this strong attraction was caused by large surface tension of the cyanobacteria owing to its intact, spherical shape. When the AFM tip gently indents the surface at every tapping cycle (so-called repulsion-dominant regime), the phase is maintained to be less than 90°. Within this regime, phase is known to be sensitive to the viscoelastic stiffness of the surface: the increase in phase qualitatively indicates that the mechanical stiffness is reduced. Comparing the phase values within the repulsive dominant regime where phase is less than 90°, one can see that the stiffness of the cyanobacteria is reduced with the phage infection.

### 2.3. Morphology of Ma-LEP

**Figure 3:**
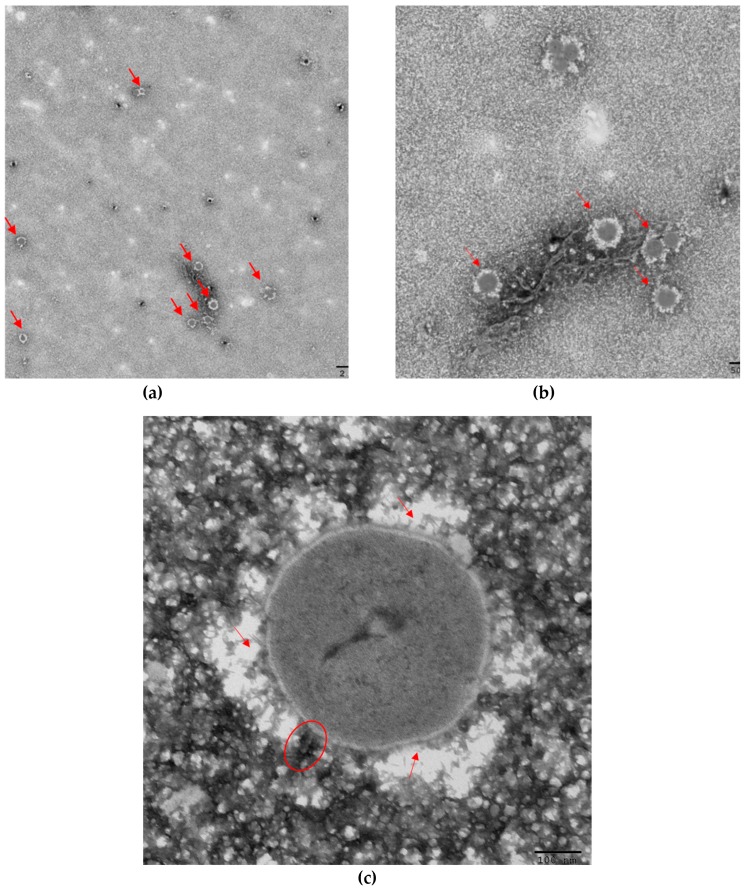
TEM images of repeating cyanophages (red arrows in **a**,**b**) with short tail (red circle in **c**) and special capsids (red arrows in **c**).

Ma-LEP was taken for TEM imaging to visualize structure of Ma-LEP (Figure 3). Multiple short-tailed viruses (~300 nm) were observed (Figure 3a). Interestingly, the structure of capsids (Figure 3c) looked similar to the knob-like proteins found in marine phage *Syn5* [7], leading to a deeper investigation of this novel structure (see more details in Section 2.4).

### 2.4. Genetic Characterization

**Figure 4:**
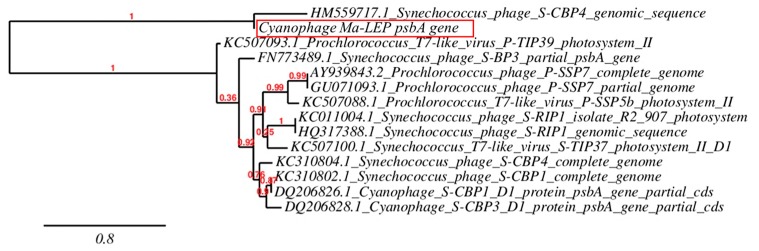
Phylogenic tree of cyanophage Ma-LEP based on the sequence of *psbA* gene (accession number: MK765681). The horizontal lines show genetic distance and the bar at the bottom of the figure denotes distance.

For genetic characterization of the isolated cyanophage, multiple signature genes from previous studies were tested (Table S2) and only one photosynthesis-related gene was present. The core gene *psbA*, originated from the cyanobacteria photosystem II core protein D1, was detected by PCR and yielded a 582-base pair (bp) gene fragment (accession #: MK765681). The sequence showed 79% identities with the one in *Synechococcus* (a major marine cyanobacteria genera, but also inhabits in freshwater) cyanophage, which suggests a potential common host range (Figure 4). One potential hypothesis is that cyanophages may take advantage of their own replications by regulating photosynthetic capacity of the hosts [12,33,34,35]. It was also reported that the *psb* gene serves to protect host cells from light stress, which induces excessive oxidative species and damages to the photosynthetic complex [36]. In addition, through gene transfer from host to phages, the *psbA* gene may be shared by various cyanophages and can serve as a ubiquitous indicator of co-evolution [29,37]. Interestingly, the freshwater cyanophage Ma-LMM01 isolated in Japan does not have *psb* gene [24], but contains a phycobilisome (a major photosynthesis complex, especially in *Synechococcus*) degradation gene (*nblA*). Other freshwater tail-less cyanophages were also known to carry the *nblA* [20,21]. However, *nblA* gene was absent in our cyanophage Ma-LEP (data not shown).

As TEM images showed, a “knob-like protein”, a unique structure of capsid protein in cyanophage *Syn5*, seemed to be present on Ma-LEP’s capsid [7,8]. This special protein may be a stabilizer for viral capsids. Instead of “sewing” the capsomere together as stabilizing proteins in other viruses do, it displays a distinctive diagonal positioning on the hexametric capsomeres of mature *Syn5* capsid and “breaks” the symmetric structure of viral capsids [7]. Three potential genes (*gp55, gp57, gp58*) encoding this protein were identified and sequenced, but there have been no additional reports of its presence in other cyanophages. In this study, using self-designed *gp58* primers yielded a ~300bp-fragnent, which is similar to the *gp58* of Syn5 [9] (100% identities; accession number: MK765680). This result further confirmed the presence of this novel protein and similar evolutionary roots of the two cyanophages. It can be predicted that the cyanophage Ma-LEP may possess similar arrangements of capsid proteins, but additional details are needed using more advanced imaging techniques. However, no phylogenic tree can be created due to its rare presence.

In summary, this is the first study to utilize the phase image data from AFM and to visualize the changing mechanical stiffness of *M. aeruginosa* membranes after cyanophage infection. The short-tailed cyanophage, named as Ma-LEP, from Lake Erie can infect bloom-forming toxic cyanobacteria, *Microcystis aeruginosa*, and negatively affect the host’s photosynthesis and growth. Ma-LEP contains two signature genes, *psbA* and *gp58*, but more genome data is needed in a future study. The results from this study provide an insight into *Microcystis*-cyanophage interactions in bloom dynamics and a potential application of cyanophage for bloom control in adequate settings.

## 3. Materials and Methods

### 3.1. Water Sample Collection, Concentration, and Screening of Lytic Cyanophages

**Figure 5:**
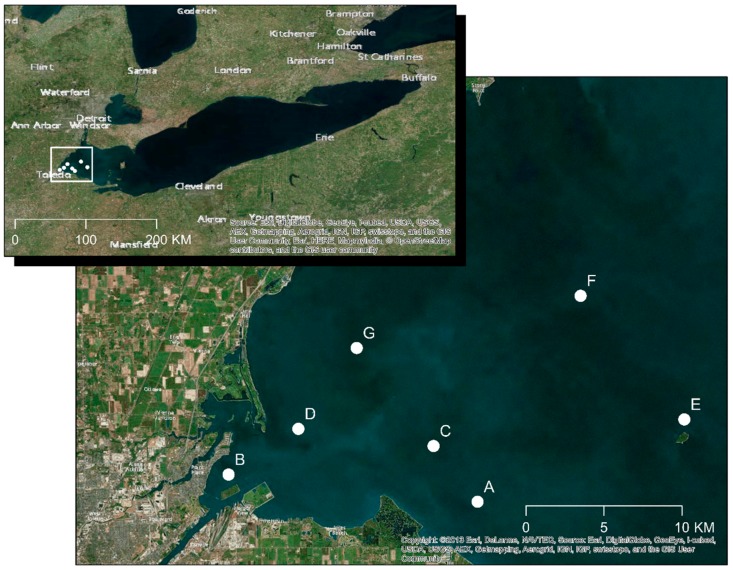
Sampling locations western Lake Erie.

Water samples were collected from western Lake Erie from 2013 to 2015 at seven different locations (Figure 5). The map was created using ArcGIS for Desktop 10.2 (Esri, Redlands CA, USA). Viruses in water samples were collected and concentrated using cation-coated filter methods [38]. Briefly, 500 mL of water was passed through an Al^3+^-coated 0.45µm filters (EMD Millipore Filter, SIGMA-ALDRICH Co., St Louis, MO, USA), and viruses captured by the filter were eluted into 10 mL of 1.0 mM NaOH (pH = 10.8) after rinsing with 200 mL of 0.5 mM H_2_SO_4_ (pH 3.0). The eluate was neutralized with 100 μL of 50 mM H_2_SO_4_ (pH 1.0) in 10 mL of 1× Tris-EDTA buffer (pH 8.0), and concentrated by centrifugation at 3000 g for 10 min (twice) using a Centriprep™ YM-50 Filter (4310 centrifugal concentrator regenerated cellulose 50 kDa NMWL, EMD Millipore, Billerica, MA, USA). Fifty microliters of each concentrate were inoculated into an optimized well-assay containing 100 μL of *Microcystis aeruginosa* culture, which was originally isolated from Lake Erie (see below section), and then incubated at room temperature with a 12 h light cycle for 2 days to screen for lytic cyanophages by measuring the OD at 680 nm.

### 3.2. Host Bacteria: Microcystis aeruginosa

*M. aeruginosa* was isolated from Lake Erie using BG-11and CT media with agarose method [39]. *M. aeruginosa* was first identified by targeting PC-IGS (phycocyanin intergenic spacer) and microcystin-producing *mcyA* and *mcyE* genes [40]. Furthermore, *M. aeruginosa* was identified by following Nanopore’s protocol (1D genomic DNA by ligation protocol). For that, ligation sequencing kit 1D (SQK-LSK108, Oxford Nanopore Technologies, Oxford, UK) was used and then real-time sequencing technique, MinION (Oxford Nanopore Technologies, Oxford, UK) [41,42] was used. Briefly, bacterial DNA was extracted with QIAamp genomic DNA kit (Qiagen, Valencia, CA, USA). Concentration of the extracted DNA (~1 µg) was measured with Qubit 3.0 fluorimeter (Thermo Fischer Scientific, Waltham, MA, USA). For fragmented DNA repair and end-repaired DNA, NEBNext FFPE repair Mix and NEBNext End repair/dA-tailing Module (New England BioLabs Inc., Ipswich, MA, USA) was used, respectively. After DNA purification with AMPure XP beads (Beckman Coulter, Brea, CA, USA), the sample was loaded on the SpotON flow cell (Oxford Nanopore Technologies, Oxford, UK). A 72 h sequencing protocol was applied using the Nanopore sequencing software, MinKNOW (v1.10.23, 2017, Oxford Nanopore Technologies, Oxford, UK), in order to collect electronic signal data. Oxford Nanopore Technologies provides a bioinformatics tool, which is a cloud-based EPI2MEAgent platform. Figure S1 shows the identification result of the *M. aeruginosa* from Lake Erie.

### 3.3. The Effects of Cyanophage Infections on Microcystis aeruginosa

Cyanophage concentrates (four independent sets) or autoclaved cyanophage concentrates (as a control) were inoculated into a *Microcystis aeruginosa* culture (host) at a ratio of 1:100 by volume, then incubated at room temperature with a 12 h light cycle for 2 weeks. The optical density at 680 nm, and concentrations of phycocyanin and chlorophyll-a were measured to monitor the dynamic changes of the host, using a spectrophotometer (4001/4 Spectronic Unicam, Moyer Instruments, Inc. Tamaqua, PA, USA) or an AquaFluor Handheld Fluorometer (8000-010 Turner designs, San Jose, CA, USA) with units of phycocyanin and *in vivo* chlorophyll-a (details can be found at http://www.turnerdesigns.com/t2/doc/spec-guides/998-8081.pdf) [43]. DNA of *Microcystis aeruginosa* was extracted using ZR fungal/bacteria DNA MicroPrep™ Kit (Zymo Research Corp, Irvine, CA, USA) following the manufacturer’s instruction. The quantification of the toxin-producing *Microcystis* by targeting *mcyE* was performed using CFX96 TouchTM Real-time PCR Detection System (Bio-Rad, Hercules, CA, USA) in duplicate (Table S2). The total volume of PCR reaction was 20 μL containing 2 μL extracted DNA, 0.5 μM of each primer, 0.125 mM probe, 10 uL of TaqMan^®^ Universal PCR Master Mix II (ThermoFisher Scientific, Grand Island, NY, USA). The microcystins were measured by Abraxis microcystins/nodularins-ADDA ELISA kit (Abraxis LLC, Warminster, PA, USA) following EPA method 546 [44].

### 3.4. Atomic Force Microscopy

Gelatin-coated mica (gelatin from porcine skin, Sigma, CAS# 9000-70-8, St. Louis, MO, USA; PELCO^®^ mica sheet, Ted Pella Inc., Redding, CA, USA) was prepared as previous research described [45]. One mL of cyanophage propagation from Method 3.5 was centrifuged at 2320× *g* for 2 min (Eppendorf centrifuge 5415R with F45-24-11 rotor, Hauppauge, NY, USA), and the supernatant was discarded. The pellet was mixed with 10 µL of 2.5% of Glutaraldehyde to fix overnight at 4 °C, which was then applied on the gelatin-coated mica, using a pipette tip and rested for 10 min. The mica slides were washed by sterilized deionized water to remove extra propagation material and dried at aseptic atmosphere for imaging. Imaging was performed in tapping mode of a commercial AFM (MFP-3D infinity from Asylum Research) equipped with silicon cantilever (natural frequency ~70 kHz, spring constant ~2 N/m, AC240 from Asylum Research).

### 3.5. TEM

Ten microliters of the cyanophage propagation was mixed with 10 μL of 5% of Glutaraldehyde to fix the samples. Carbon/Formvar-coated copper grids were glow discharged in a PELCO easiGlow™ discharge unit. Ten µl of drop sample were incubated on the grid for 5 min, and then incubated with a drop of 1% uranyl acetate for 30 s after removing excess sample. Grids were imaged in a FEI Tecnai™ G2 Biotwin TEM (ThermoFisher Scientific, Hillsboro, Oregon, USA) at 80 kEV and images captured using an AMT camera and software (R5.6, 2017, Thermo Fisher Scientific, Woburn, MA, USA).

### 3.6. Targeting Viral Genes Using PCR

The viral DNAs from cyanophage concentrates were extracted using PowerViral Environmental DNA Isolation Kit (MO BIO, San Diego, CA, USA). Primers of *gp55*, *gp57* and *gp58* (knob-like protein genes) were generated from sequences of corresponding genes using NCBI Primer Blast [38,39]. Other PCR primers, including *psbA*, and PCR conditions were described in Table S1. PCR amplicon were checked by 2% agarose gel at 50 V for 30 min and purified by QIAquick PCR Purification Kit (Qiagen, Germantown, MD, USA) following manufacturer’s instruction. Sequencing was performed by BigDye^®^ Terminator Cycle Sequencing combined with 3730 DNA Analyzer (ThermoFisher Scientific, Grand Island, NY, USA) and identified by NCBI BLASTn [40]. The phylogenic analyses of cyanophage Ma-LEP based on the sequence of psbA gene (accession number: MK765681) was carried out using a software from the PhyML program, which is available at www.phylogeny.fr [45,46,47,48,49].

### 3.7. Statistical Analysis

Data were fitted into a linear regression model using SPSS Statistics for Windows, Version 24.0 (2017, IBM, Armonk, NY, USA).

## Figures and Tables

**Table 1:** Pigment production and growth of *M. aeruginosa* with or without cyanophage Ma-LEP infection as a function of time in a linear model.

Treatment	Phycocyanin		Chlorophyll-a		OD at 680 nm		Log (Gene Copy Number) ^1^	
	Slope	*R* ^2^	Slope	*R* ^2^	Slope	*R* ^2^	Slope	*R* ^2^
Control	13.10	0.93	3.45	0.84	0.04	>0.99	0.28	0.82
Cyanophage Ma-LEP	5.75 **	0.68	2.18 **	0.90	0.03 **	0.99	0.13 **	0.74

^1^ Data from day 1 to day 5 were fitted, ** *p* < 0.01.

## Supplementary Materials

The following are available online at https://www.mdpi.com/2072-6651/11/8/444/s1, Figure S1: Taxonomy tree at the genus level and classification of *Microcystis* with read numbers. Figure S2: Induction of lysogenic cyanophage Ma-LEP by UV irradiation. Figure S3: Microcystin concentrations with or without lysogenic cyanophage Ma-LEP infection (no significant difference). Table S1: Pigment production and growth of *M. aeruginosa* with or without lysogenic Cyanophage Ma-LEP infection after UV irradiation as a function of time in a linear model. Table S2: PCR conditions and primer sequences used in this study (Reference [50] is cited in the Supplementary Materials).
