# Supplementary material for: Characterization of Cyanophages in Lake Erie: Interaction Mechanisms and Structural Damage of Toxic Cyanobacteria
Source: Toxins (Basel). 2019 Jul 26;11(8):444. doi: 10.3390/toxins11080444 (PMC6722964; doi:10.3390/toxins11080444)
Supplement: Supplementary file 1 [file toxins-11-00444-s001.pdf]

Supplementary Materials: Characterization of Cyanophages in Lake Erie: Interaction Mechanisms and Structural Damage of Toxic Cyanobacteria

Xuewen Jiang, Chanhee Ha, Seungjun Lee, Jinha Kwon, Hanna Cho, Tyler Gorham and Jiyoung Lee

Results and Discussions

UV-Induced Activation of the Prophage

Ma-LEP also showed lysogenic activity during continuous culturing under lab conditions when appropriate dose of UV light was applied. Figure S2 shows the dynamic changes in *M. aeruginosa* population and photosynthesis capacity. At first, two cycles of UV irradiation did not show any effects on host growth, but after the third UV treatment, the host rapidly entered the lytic stage and host cells started being lysed. In this study, 23.58 mJ/cm^2 of UV intensity was sufficient for the cyanophage to activate cell lysis. The OD at 680 nm slightly decreased in one week. In contrast, the uninfected cells steadily increased over time (Figure S2a, e). The photosynthetic pigment production also showed a dramatic decrease after UV treatment (Figure S2c, d). However, *M. aeruginosa* population slightly increased at the beginning, then was followed by a decrease at day 9 (Figure S2b), by measuring the *mcyE* gene using qPCR. This may be explained by the delay in gene disintegration, due to the stability of DNA as mentioned in the main text.

The raw data collected after full dose of UV was given (Figure S2 from day 5 to day 11) and were fitted in linear models by IBM SPSS Statistics for Windows (Version 24.0). Table S2 describes the growth of pigment production rate (slope) and correlation coefficient (R^2) of each linear model. The phage-infected *M. aeruginosa* showed a significant decrease in growth rate by measuring OD at 680 nm ($p < 0.01$), but the other three models were not statistically significant. In the lysogenic cycle, the viral *psbA* may be supplementary to host photosynthesis to provide essential energy and material for host growth. Therefore, phycocyanin and chlorophyll were increased before the induction (Figure S2c,d, days 1–5). With a low dose of UV irradiation, expression of the viral *psbA* gene may be a major repairing pathway in the photosystem; however, photo-stress caused by UV is irreversible via this mechanism, Ma-LEP starts to be lytic to save and spread its own genes. Therefore, the pigment concentration dramatically decreased in both groups due to severe damage to the photosynthesis capacity of *M. aeruginosa*. However, more studies are needed to identify the mechanisms of this phenomenon.

Material and Methods

UV-Induced Activation of the Prophage

One milliliter of propagated phage (four independent sets) or sterilized DI water was inoculated into 300 ml of *M. aeruginosa* (OD_{680nm} ~0.05) and cultured as described above. Prophage cocktail was treated with UV light with three cycles of 10 s irradiation following 10 s resting every day for three days. The total dose was 23.58 mJ/cm^2 following the equation below:

$$\text{UV dose} = \text{intensity} \times \text{exposure time} \quad (1)$$

The effects on *M. aeruginosa* were measured with the same methods described in the Materials and Methods (Section 3.3).

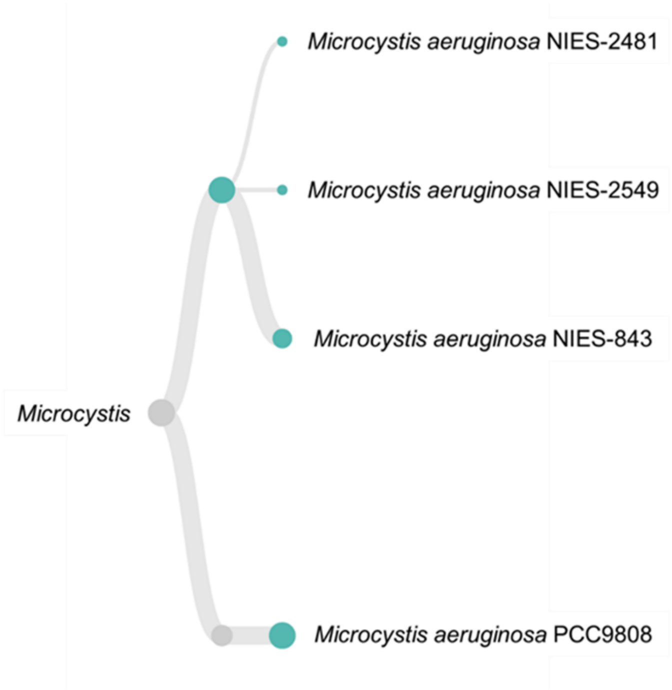

Taxon	Reads
Microcystis aeruginosa PCC9808	60,056
Microcystis aeruginosa NIES-843	36,115
Microcystis aeruginosa	14,972
Microcystis aeruginosa NIES-2549	4665
Microcystis aeruginosa NIES-2481	4052

Figure S1. Taxonomy tree at the genus level and classification of *Microcystis* with read numbers. Average sequence length was 4572 bases and average quality score was 11.21. A total of 99.35% genes of the total classified genes (120,644) were related with *Microcystis aeruginosa*.

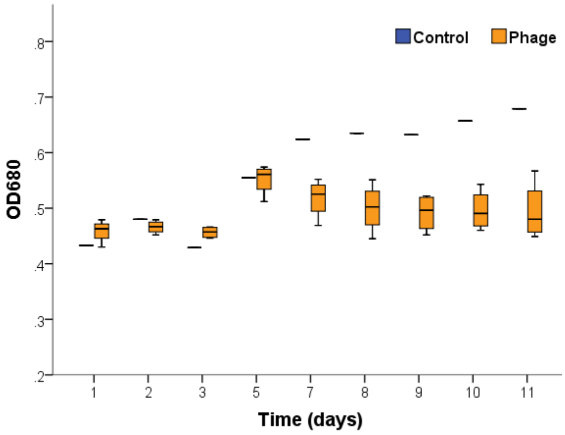

(a)

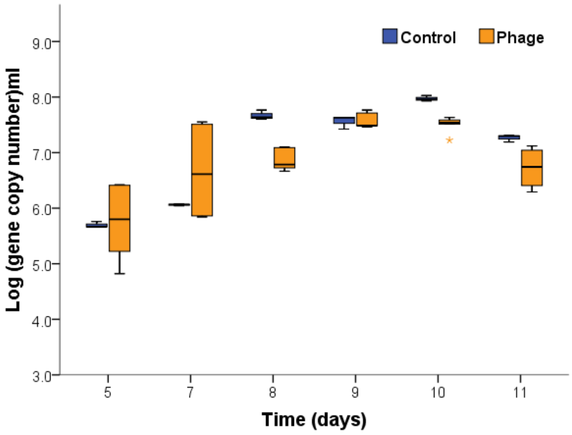

(b)

(c)

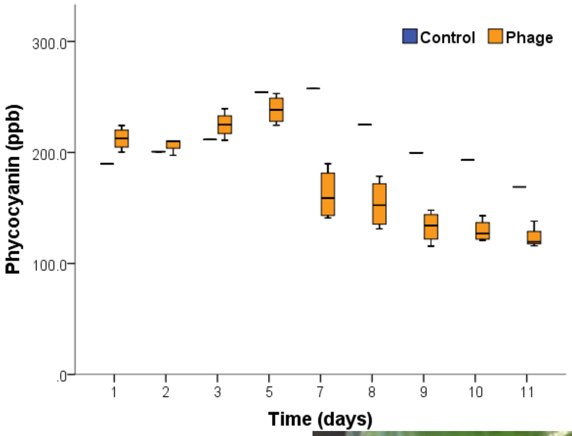

(d)

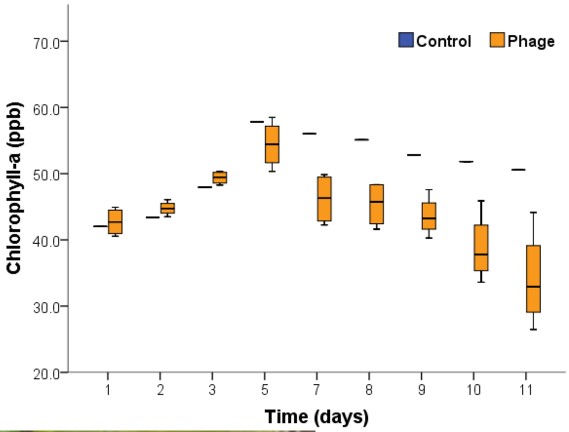

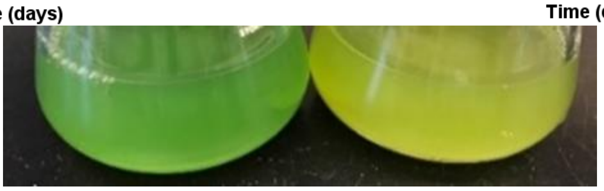

(e)

Figure S2. Induction of lysogenic cyanophage Ma-LEP by UV irradiation. Infected *M. aeruginosa* was irradiated by UV light on day 1, 2, and 4 and monitored for additional week. (**a**) Optical density (OD) at 680 nm; (**b**) gene copy number of *M. aeruginosa* in log scale; (**c**) the concentration of phycocyanin; (**d**) the concentration of chlorophyll-a; (**e**) normal *M. aeruginosa* (left) and infected by cyanophage Ma-LEP (right) after UV treatment

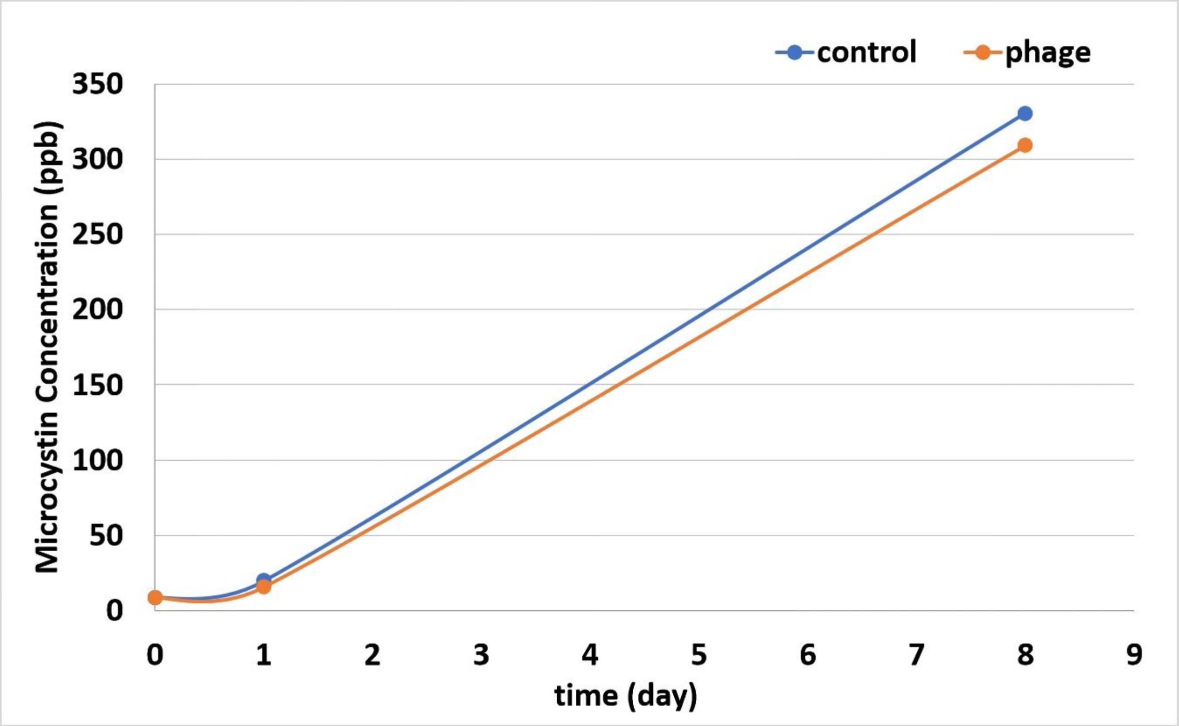

Figure S3. Microcystin concentrations with or without lysogenic cyanophage Ma-LEP infection (no significant difference).

Table S1. Pigment production and growth of *M. aeruginosa* with or without lysogenic Cyanophage Ma-LEP infection after UV irradiation as a function of time in a linear model.

Treatment	Phycocyanin		Chlorophyll-a		OD680nm		Log (gene copy number) [1]	
	Slope	*R*2	Slope	*R*2	Slope	*R*2	Slope	*R*2
Control	−15.44	0.94	−1.26	0.99	0.02	0.96	0.35	0.80
Cyanophage Ma-LEP	−18.41**	0.87	−3.15	0.83	−0.01**	0.48	0.25	0.62

[1] data from day 5 to day 11 were fitted, ** $p < 0.01$.

Table S2. PCR conditions and primer sequences used in this study.

Genes		Function	Primers	Condition	References
Mydoviridae	*g20*	Capsid assembly protein	F:CATWTCWTCCCAHTCTTC<br>R:SWRAAATAYTTICCRACRMAKGGATC	Denaturation at 95 °C for 5 min, 35 cycles of denaturation at 95 °C for 1 min, annealing at 51–60 °C for 30 min, and extension at 72 °C for 1 min, and a final extension at 72 °C for 10 min.	[28,29]
	g91	Tail sheath	F:AGYGAGTTYCGCCTTAHTGT<br>R:GRTGAYTGRCGTACYARRGC		
	mcp	Major capsid protein	F:GTTCCTGGCACACCTGAAGCGT<br>R:CTTACCATCGCTTGTGTCGGCATC		
	nblA	Phycobilisome degradation protein	F:GTGAGTGCCATTCCTGC<br>R:TCTTCTTGATGATAGCCGC		[37]
	nblA		F:GGAGAATTCCAAATGGATACAGATTC<br>R:GTGTAAGCTTTTAGGCGGGGT		[20]
	psbA	Photosynthesis protein D1	F:TAYCCNATYTGGGAAGC<br>R:TCRAGDGGGAARTTRTG		[50]
	pol	Polymerase	F:CCNAAYYTNGSNCARGT<br>R:TCRTCRTGNAYRAANGC		
Podoviridae	*gp55*	Knob-like proteins on capsid	F:TGGGTGGTTACTTCTTCCTT<br>R:AATCTCGTCGATGTCAGACT		This study
	gp57		F:TACTACCAATGATGCTGCTC<br>R:GATCCGCCTTATGTAGATCC		
	gp58		F:CAAGGCTCTGAACGAATCC<br>R:GGTGAGAGCAGTGATGTTG		
Microcystis	*mcyE*	Microcystin production	F:AAGCAAACTGCTCCCGGTATC<br>R:CAATGGGAGCATAACGAGTCAA<br>Probe:6FAM-CAATGGTTATCGAATTGACCCCGGAGAAAT-TAMARA	50 °C for 2 min, 95 °C for 10 min, 40 cycles of 95 °C for 30 s, 62 °C for 1 min.	[40]
